# Modelled Physical Activity Benefits and PM_2.5_ Risks in Two Urban Cyclists

**DOI:** 10.3390/toxics14090836

**Published:** 2026-09-21

**Authors:** Anna Mainka, Malwina Mainka

**Affiliations:** 1 Department of Air Protection, Faculty of Energy and Environmental Engineering, Silesian University of Technology, Konarskiego 22B, 44-100 Gliwice, Poland; 2 Faculty of Biomedical Engineering, Silesian University of Technology, Roosevelta 40, 41-800 Zabrze, Poland

**Keywords:** urban transport, active commuting, bicycle, city center, public health, Poland

## Abstract

The analysis evaluated the modelled balance between physical-activity benefits and PM_2.5_ -related mortality risks using previously published monitoring data from 162 repeated cycling trips by two commuters in Gliwice, Poland. Each trip defined a hypothetical long-term pattern repeated five days per week, compared with resting for the same duration at the concurrent stationary reference concentration. Observed concentrations and durations were combined with assumed ventilation and published dose–response functions. The analysis propagated epidemiological coefficient uncertainty and alternative ventilation assumptions into net relative risk (*RR_NET_*) and examined illustrative sensor sensitivity and trip-specific break-even concentrations. Under central fixed-ventilation assumptions, median *RR_NET_* was 0.840, corresponding to a modelled 16.0% relative risk reduction. The simulated dataset median was 0.839 (95% uncertainty interval 0.792–0.892) when epidemiological coefficients varied. Alternative ventilation scenarios also yielded favourable conditional balances. Personal-concentration changes of ±10% altered the sign of the dose-equivalent increment for 12 trips without producing central *RR_NET_* ≥ 1. The median modelled break-even concentration was 196.7 µg/m^3^ with other assumptions fixed. These estimates are not observed health effects, population effects or universal pollution thresholds. Uncalibrated optical measurements and the two-person sample constrain interpretation; larger studies with sensor validation and measured ventilation are needed.

## 1. Introduction

Urban cycling is increasingly promoted as a sustainable mode of transport and a practical means of incorporating regular physical activity into daily mobility. Health-impact assessments comparing cycling with motorized transport have generally concluded that the health benefits associated with increased physical activity outweigh the additional risks related to air pollution exposure under most urban conditions [1,2,3,4,5,6,7]. These studies established the conceptual basis for evaluating the trade-off between the beneficial effects of active travel and the adverse effects of inhaled pollutants. However, the magnitude of the estimated net benefit depends strongly on assumptions regarding background air pollution, exposure during cycling, ventilation, dose–response relationships, and cycling duration [4,7].

An important component of this balance is the health benefit associated with habitual cycling. Cycling for transport can contribute substantially to recommended levels of moderate-intensity aerobic physical activity and can be integrated into everyday travel without requiring separate time for structured exercise [8]. Current WHO guidance recommends 150–300 min of moderate-intensity aerobic physical activity per week for adults and recognizes transport-related activity, including cycling, as a means of achieving these recommendations [8,9]. Epidemiological and intervention studies associate cycling and active commuting with lower risks of premature mortality, cardiovascular disease, diabetes and other non-communicable disease outcomes, as well as improved cardiorespiratory fitness and a more favourable cardiovascular profile [10,11,12,13,14]. These benefits constitute the principal benefit component in quantitative risk–benefit assessments of urban cycling.

The potential countervailing risk arises because physical activity increases pulmonary ventilation and therefore the amount of air—and pollutants—inhaled during a journey. Consequently, pollutant concentration alone may not adequately characterize exposure during active commuting. Cyclists may experience concentrations comparable to or lower than those encountered in motorized transport while receiving a higher inhaled dose because of increased ventilation [15,16,17]. Studies incorporating travel duration, respiratory ventilation and exercise intensity have therefore emphasized the distinction between external concentration, inhaled dose and the amount of particulate matter potentially deposited in the respiratory tract [18,19,20,21]. This distinction is particularly relevant for PM_2.5_ because exercise-related increases in ventilation may enhance pollutant uptake.

Exposure during cycling is also highly heterogeneous in space and time. Traffic intensity, intersections, route characteristics, cycling infrastructure, time of day and local emission sources can substantially modify personal PM exposure [22,23,24,25,26,27,28,29]. Recent real-world studies further demonstrate that estimates of cyclists’ pollutant burden can change when trip duration, exercise intensity, ventilation or individual trajectories are incorporated into exposure assessment [30,31,32,33]. Concentration-based and dose-based metrics do not necessarily classify cyclists in the same way, reinforcing the need to account for physiological variability when assessing exposure during active travel [33]. Spatial inequalities in cycling-related environmental risks may also affect the distribution of benefits across populations [34,35], while perceived or actual air pollution can influence cycling behaviour itself [36].

Whether air pollution substantially attenuates the long-term health benefits of physical activity remains less clear. Prospective studies conducted in China suggest that the protective associations of active commuting may become weaker at relatively high long-term PM_2.5_ concentrations [37,38], whereas recent UK evidence indicates that cycling can remain associated with lower cardiovascular risk at substantially lower European pollution levels [39]. Systematic and mapping reviews similarly indicate that air pollution can modify some health effects of active mobility but does not support a simple universal pollution threshold above which physical activity becomes harmful [40,41]. Studies of physical activity more broadly generally show persistent health benefits across a range of pollution exposures, although attenuation may occur with increasing PM_2.5_ levels, depending on population, outcome, activity intensity and exposure conditions [42,43,44,45]. Recent meta-analyses support an overall mortality benefit of physical activity in polluted environments while also suggesting that the magnitude of protection may decline at higher PM_2.5_ concentrations [46,47].

Modelling studies [4] indicate that the health benefits of cycling generally outweigh the risks associated with air pollution, except under unusually high pollution levels or prolonged cycling durations. However, these assessments were primarily based on background concentrations and assumed exposure ratios rather than directly measured personal exposure. Subsequent evidence indicates that the balance may vary considerably with population characteristics, pollution level and the methods used to characterize both physical activity and air pollution exposure [38,41,42,43,44,45,46,47].

This methodological distinction is central to the present study. Most previous risk–benefit assessments have relied on background or residential PM_2.5_ concentrations and assumed relationships between ambient concentrations and cyclists’ exposure [1,2,3,4,5,7,41], whereas real-world exposure studies demonstrate substantial trip-to-trip variability and show that concentration alone may not adequately represent the pollutant burden experienced during cycling [18,19,20,21,29,30,31,32,33]. Personal measurements obtained during actual bicycle journeys can reduce this uncertainty by incorporating the concentrations encountered in the cyclist’s immediate microenvironment.

The aim of this analysis was to evaluate a PM_2.5_–physical-activity benefit–risk model using the cycling subset of a previously published commuting dataset [30]. Its contribution is the joint propagation of epidemiological coefficient uncertainty and alternative ventilation assumptions through the final relative-risk estimates, together with deterministic sensor sensitivity, sign-change and trip-specific break-even analyses. We retain the observed variation among 162 trips while restricting interpretation to the commuting patterns of two participants. The study neither estimates population health effects in Gliwice nor tests the effects of replacing another transport mode with cycling.

## 2. Materials and Methods

### 2.1. Study Design and Cycling Dataset

This analysis used the commuting dataset reported by Mainka et al. [30], collected in Gliwice, Upper Silesia, Poland, between January and November 2022. December would also be classified as part of the heating season; however, the original monitoring dataset contained no cycling observations from that time. No new exposure measurements or health outcomes were collected for this analysis. The original campaign monitored two participants using different transport modes. The present bicycle subset comprises Participant 1 (male; 91 trips) and Participant 2 (female; 71 trips). Participant identity and sex are completely confounded. Sex is used only to document the assigned ventilation assumptions, and participant comparisons are descriptive.

We selected the 162 records classified as bicycle trips and retained the trip-level personal PM_2.5_ concentration, duration, distance and concurrent reference concentration used in the central dose model. There were 78 heating-season and 84 non-heating-season trips. The heating season is defined as October–March, including December; no December observations were available because monitoring ended in November. April–September constitutes the non-heating season. Mean cycling duration was 34.6 ± 23.5 min in the non-heating season and 38.8 ± 20.7 min during the heating season, while the corresponding mean trip distances were 3.6 ± 4.0 km and 5.6 ± 4.2 km, respectively. These durations describe the two observed commuters and are not a representative distribution of cycling times in Gliwice.

Each record defines a separate hypothetical exposure pattern: its duration and exposure conditions are assumed to recur on five days per week over the long term. The 162 patterns are not summed as though all trips were performed by one person each week. Measured quantities are personal PM_2.5_ concentration and the recorded temporal and spatial trip information. Ventilation, inhaled dose, dose-equivalent concentration and all health-effect estimates are modelled quantities.

**Figure 1:**
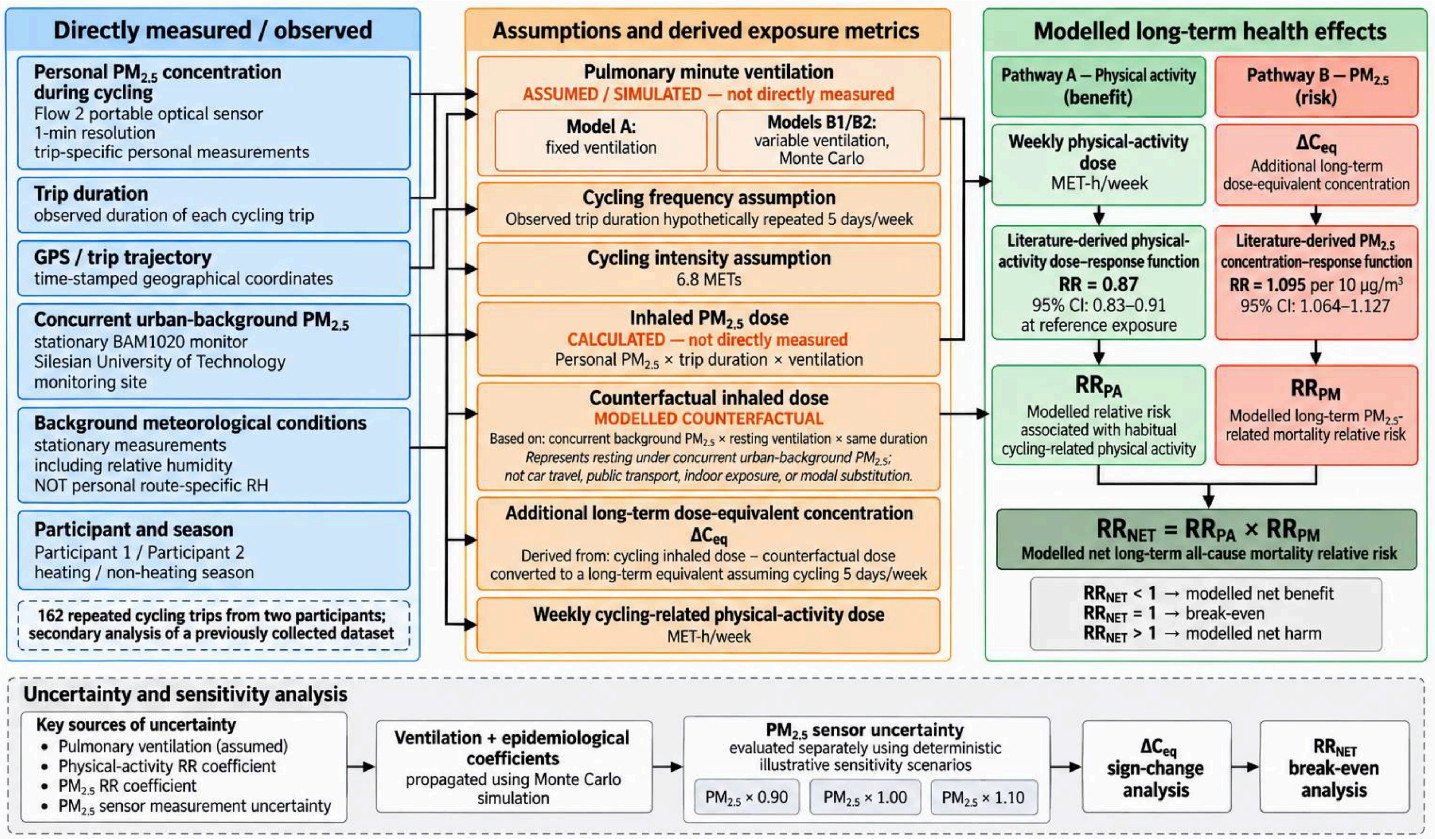
Framework distinguishing observed data from model-derived health estimates.

Figure 1 summarizes the observed inputs, assumed parameters, modelled benefit–risk pathways, and uncertainty and sensitivity analyses.

Uncertainty intervals reflect the propagated epidemiological coefficients and specified ventilation assumptions. The ±10% personal-concentration scenarios are illustrative and do not quantify device-specific measurement error or total model uncertainty. No health outcomes were directly measured.

### 2.2. Personal PM_2.5_ Exposure During Cycling

The original monitoring campaign used one Flow 2 portable optical sensor (Plume Labs, Paris, France), shared by the two participants during their respective trips. It recorded PM_2.5_ at 60 s intervals [30]. Participant identity and timing linked each series to the appropriate participant. The smartphone application recorded time-stamped geographical coordinates. The present secondary analysis uses the processed trip summaries and does not repeat the acquisition or reconstruction of minute-level trajectories.

No study-specific gravimetric calibration, device-specific error distribution or quantitative relative-humidity (RH) correction was available for the Flow 2 measurements. Optical mass estimates can depend on aerosol composition and water uptake; correlations reported in other studies do not establish the accuracy of this unit. A chamber evaluation found substantial underestimation for incense-generated particles [48], illustrating that the direction and magnitude of error depend on the conditions. Neither that experiment nor the manufacturer information reported in the earlier publication [30] provides an applicable correction equation. We therefore retain the supplied concentrations and explicitly examine illustrative sensor scenarios, while acknowledging that their range may not encompass the actual error.

The earlier study used a custom Python 3 script to identify continuous time-stamped tracks, match pollution measurements to those tracks and calculate distances using GeoPy [30]. That publication also describes interpolation of some missing minute-level records. The present secondary analysis starts from the supplied processed trip means and performs no additional imputation. Consequently, it cannot determine the frequency or influence of any upstream interpolation without the original minute-level records and processing code. Trip identifiers were cross-matched between the reference model and the processed bicycle records using concentration, duration and distance, with participant and season checks.

The counterfactual concentration was the concurrent trip-specific PM_2.5_ value from the Silesian University of Technology mobile laboratory operating at a fixed location, using a BAM1020 monitor (Met One Instruments, Grants Pass, OR, USA). Meteorological measurements were recorded with a WS500 (Lufft, Fellbach, Germany) weather station [30]. The values used in the model match the mobile-laboratory column of the supplied dataset. They are treated as a stationary reference for the selected counterfactual, not as a measurement of the entire cycling route or evidence of citywide background representativeness. Measurements at this separate location do not constitute collocated calibration of the personal sensor.

Walking, motorized-travel and stop records were excluded from the secondary model. All 162 bicycle records had the required trip-level concentration, duration and counterfactual inputs. Additional RH stratification was restricted to 150 trips with numeric concurrent RH. The remaining 12 trips were retained in the principal analysis and reported separately in the RH analysis.

Personal and stationary concentrations describe different locations and need not have the same seasonal pattern. We therefore present personal concentrations by participant and season, with all trips visible in Section 3.1. Possible contributions from route, timing, local sources and sensor response are considered in the Discussion; the present records do not identify their separate effects.

### 2.3. Inhaled PM_2.5_ Dose

The inhaled dose of PM_2.5_ during cycling was estimated by integrating personal PM_2.5_ exposure with commuting duration and pulmonary minute ventilation. This approach was selected because pollutant concentration alone does not adequately characterize the respiratory burden during active commuting. Inhaled dose is determined jointly by the concentration encountered, the duration of exposure, and the volume of air inhaled, with pulmonary ventilation becoming particularly important during cycling because of the increased metabolic demand associated with physical activity [19,30,49]. Borghi et al. [49] identified exposure concentration, residence time, and pulmonary ventilation rate as the principal determinants of inhaled pollutant dose, particularly in transport microenvironments and during active commuting.

Each of the 162 cycling trips was treated as an individual observation in the dose model. For trip, the inhaled PM_2.5_ dose was calculated according to the approach previously applied by Mainka et al. [30]:

(1) $${ID}_{i}=\bar{C_{i}}\times t_{i}\times {VE}_{s}$$

where *ID_i_* is the inhaled PM_2.5_ dose during trip (µg), $\bar{C_{i}}$ is the mean personal PM_2.5_ concentration measured during that trip (µg/m^3^), *t_i_* is cycling duration (min), and *VE_s_* is the sex-specific pulmonary minute ventilation rate (m^3^/min). This formulation is consistent with the previous analysis of the Gliwice commuting dataset, in which inhaled dose was defined as a function of measured pollutant concentration, exposure duration, and mode-specific ventilation rate.

The dose normalized to travelled distance was calculated as:

(2) $${ID}_{i,km}=\frac{{ID}_{i}}{d_{i}}$$

where *d_i_* is the distance travelled during trip *i* (km), and *ID_i_*_,*km*_ is expressed in µg/km.

#### 2.3.1. Model A: Fixed Ventilation Rate

Model A retained the ventilation rates used in the original dose calculations [30]. For cycling, minute ventilation was assumed to be *VE* = 0.0573 m^3^/min = 57.3 L/min for Participant 1 and *VE* = 0.0457 m^3^/min = 45.7 L/min for Participant 2.

These values were previously used to estimate pollutant inhalation during bicycle commuting in the Gliwice dataset. Model A therefore provides directly comparable dose estimates while allowing the present analysis to focus exclusively on cycling and PM_2.5_.

The use of a fixed ventilation rate nevertheless represents an important simplification. Actual ventilation during cycling varies according to exercise intensity, speed, route characteristics, individual physiology, sex, and bicycle type. Lathouwers et al. [19] demonstrated under real-world cycling conditions that respiratory ventilation varied with sex, cycling mode, speed, and elevation gain and that these differences translated directly into differences in inhaled pollutant dose. Consequently, uncertainty associated with ventilation was explicitly examined in a second model.

#### 2.3.2. Alternative Ventilation Scenarios

Alternative ventilation scenarios were specified using the commuting framework discussed by Oneda et al. [21] in their modelling of PM_2.5_ deposition during urban bicycle commuting. In their first scenario (B1), intended to represent real commuting conditions, minute ventilation ranged from 11.6 to 29.5 L/min for Participant 1 and from 11.7 to 47.7 L/min for Participant 2.

B2 assigned both participants 30–40 L/min. We assumed uniform distributions within these bounds. These are stipulated trip-average scenarios, not fitted distributions of measured ventilation or estimates of population variability. We generated 10,000 trip-specific ventilation realizations for each alternative scenario. For trip *i* in iteration *j*, the dose calculation was:

(3) $${ID}_{i,j}=C_{i}\times {VE}_{i,j}\times t$$

The same observed trip-average concentration and duration were used in every realization. The ventilation draw *VE_i_*_,*j*_ was converted from L/min to m^3^/min before multiplication. Each simulated dose was then carried through the dose-equivalent concentration, *RR_PM_* and *RR_NET_* calculations. The scenarios therefore affect the final health-effect distributions rather than inhaled dose alone. The bounds were retained as a sensitivity framework. They were not inferred from the two participants’ concentration records and using them does not establish differences attributable to sex.

For dose summaries, we first calculated the median simulated dose for each trip and then summarized those trip-specific medians within the relevant group. For health-effect uncertainty, we also summarized the median across all 162 trips separately in every iteration, as detailed in Section 2.8.

### 2.4. Health Benefits of Cycling—Physical Activity Model

Physical-activity effects were modelled using the 0.50-power relationship adopted in the active-travel framework of Tainio et al. [4]. Kelly et al. [50] report *RR* = 0.87 (95% CI: 0.83–0.91) at 11.25 MET-h/week for this power transformation. This is the coefficient for the 0.50-power sensitivity model, rather than the linear-model estimate in that meta-analysis. Cycling intensity was fixed at 6.8 METs. For each hypothetical habitual pattern:

(4) $${PA}_{i}=6.8\times \left(\frac{t_{i}}{60}\right)\times 5$$

where *PA_i_* is the cycling-related physical-activity dose during trip *i* (MET-h/week), 6.8 is the metabolic equivalent assigned to utility cycling at a self-selected pace, *t_i_* is the cycling duration recorded during trip *i* (min/day), and 5 represents the number of days per week. The calculation does not establish that either participant actually maintained that specific pattern throughout a year.

Standardized scenarios of 15, 30, 45, 60 and 90 min of cycling per day were evaluated by replacing *t_i_* in the equation with the corresponding scenario duration. Following Tainio et al. [4], the transformed physical-activity dose was calculated as:

(5) $$X_{i}={\left(\frac{{PA}_{i}}{{PA}_{ref}}\right)}^{0.50}$$

where the reference physical-activity dose was: *PA_ref_* = 11.25 MET-h/week. *X_i_* is a dimensionless transformed physical-activity dose that expresses the cycling-related dose during trip *i* relative to the reference dose. The square-root transformation accounts for the nonlinear dose–response relationship and the diminishing marginal health benefit at increasing levels of physical activity. Thus, *X_i_* equals 1 when *PA_i_* equals the reference dose, is lower than 1 when *PA_i_* is below the reference dose and is greater than 1 when *PA_i_* exceeds the reference dose.

(6) $${RR}_{PA,i}=0.87^{X_{i}}$$

where *RR_PA_*_,*i*_ is the modelled relative risk for the hypothetical habitual activity pattern. The corresponding relative risk reduction was:

(7) $${Benefit}_{i}\;\left(\%\right)=\left(1-{RR}_{PA,i}\right)\times 100$$

The comparison assumes no cycling-related activity in the reference condition. We did not individualize the function for baseline activity, fitness or health status and did not model substitution of other physical activity. Thus, the estimated benefit describes the assumed additional cycling activity and is not a prediction of the improvement each monitored participant would experience. The common coefficient was applied to both participants. Differences between their scenario estimates arise from trip-duration inputs, not sex-specific epidemiological effects.

### 2.5. PM_2.5_-Related Health Risk

PM_2.5_-related effects were estimated relative to resting for the same duration at the stationary reference concentration. The central counterfactual dose was:

(8) $${ID}_{0,i}=C_{0,i}\times \left(\frac{0.61}{60}\right)\times t_{i}$$

where *ID*_0,*i*_ is the counterfactual inhaled mass (µg), *C*_0,i_ is the concurrent mobile-laboratory reference concentration (µg/m^3^), and 0.61 m^3^/h is the assumed resting ventilation [4]. This comparison does not represent indoor exposure, car travel or replacement of a real commuting alternative. Its results depend on the selected stationary site and resting scenario.

Because the concentration–response functions were derived from studies of long-term exposure, the concentration recorded during a single trip was not interpreted directly as an annual PM_2.5_ concentration. Instead, the additional trip dose was converted into a long-term dose-equivalent concentration, assuming that cycling was repeated on five working days per week:

(9) $$∆C_{eq,i}=\left(\frac{n}{7}\right)\times \left[\frac{\left({ID}_{i}-{ID}_{0,i}\right)}{V_{0}}\right]$$

where Δ*C_eq_*_,*i*_ is the long-term PM_2.5_ dose-equivalent concentration increment associated with cycling trip *i* (µg/m^3^), *n* = 5 is the number of cycling days per week, and *V*_0_ is the daily volume of air inhaled in the non-cycling reference scenario. Following Tainio et al. [4], *V*_0_ was calculated assuming 8 h of sleep and 16 h of resting activity *V*_0_ = (8 × 0.27) + (16 × 0.61) = 11.92 m^3^/day, where 0.27 and 0.61 m^3^/h are the ventilation rates during sleep and rest, respectively. *ID_i_* was obtained using the fixed sex-specific ventilation rates defined for Model A (Section 2.3.1). Consequently, the primary *RR_PM_*_,*i*_ estimates and the net health benefit–risk assessment were based on Model A. Models B1 and B2 represent stipulated trip-average ventilation sensitivity scenarios with uniform distributions. Their ventilation draws were propagated through inhaled dose, Δ*C_eq_*, *RR_PM_* and *RR_NET_*, together with uncertainty in both epidemiological coefficients, as described in Section 2.8. Sensor-related error was examined separately using illustrative deterministic concentration scenarios, while structural model uncertainty was not quantified.

For the primary analysis, the concentration–response coefficient was obtained from the updated WHO-commissioned systematic review and meta-analysis by Orellano et al. [51]. The pooled relative risk of all-cause mortality was 1.095 (95% CI: 1.064–1.127) per 10 µg/m^3^ increase in long-term PM_2.5_ exposure. The PM_2.5_-related relative risk for cycling trip *i* was therefore calculated as:

(10) $${RR}_{PM,i}=1.095^{\left(\frac{∆C_{eq,i}}{10}\right)}$$

To enable direct comparison with the model reported by Tainio et al. [4], a sensitivity analysis was performed using their concentration–response coefficient of *RR* = 1.07 per 10 µg/m^3^:

(11) $${RR}_{PM,i,Tainio}=1.07^{\left(\frac{∆C_{eq,i}}{10}\right)}$$

Only the PM_2.5_ coefficient differs in this historical sensitivity calculation. No multiplier of 2.0 is applied to the measured personal concentration.

The same PM_2.5_ coefficient was used for both participants. Participant-specific results reflect their concentration and duration records and assigned ventilation values. They do not identify biological sex effects.

Using an ambient mortality concentration–response function for an inhaled-dose-equivalent increment is a structural model assumption. Equal inhaled mass need not imply equal toxicity, deposition or biological effect across aerosols and exposure patterns. We retain this dose-based modelling convention for comparability with the active-travel framework [4], without claiming epidemiological equivalence or validation for these commuters.

### 2.6. Net Health Benefit–Risk Assessment

Trip-specific relative risks associated with physical activity (*RR_PA_*_,*i*_) and additional PM_2.5_ exposure (*RR_PM_*_,*i*_) were calculated as described in Section 2.4 and Section 2.5, respectively. The net relative risk was estimated using a multiplicative model:

(12) $${RR}_{NET,i}={RR}_{PA,i}\times {RR}_{PM,i}$$

where *RR_NET_*_,*i*_ combines the modelled physical-activity and PM_2.5_ components for the same hypothetical pattern. Multiplication assumes that their relative effects can be combined without an additional interaction term. The net relative risk reduction was:

(13) $${NetEffect}_{i}\left(\%\right)=\left(1-{RR}_{NET,i}\right)\times 100$$

The sign convention means that a lower *RR_NET_* produces a larger positive *NetEffect*. Uncertainty limits for *NetEffect* were calculated directly from its simulated distribution. The combined results were interpreted as follows:

- *RR_NET_*_,*i*_ < 1 indicates that the physical-activity benefit exceeds the PM_2.5_-related risk and cycling provides a net health benefit;
- *RR_NET_*_,*i*_ = 1 represents the break-even condition at which the physical-activity benefit is completely offset by the PM_2.5_-related risk;
- *RR_NET_*_,*i*_ > 1 indicates that the PM_2.5_-related risk outweighs the physical-activity benefit.

These classifications concern model outputs, not observed deaths, acute responses or comprehensive cycling safety.

### 2.7. Statistical and Data Analysis

All analyses were conducted at the trip level, with each of the 162 cycling trips treated as a separate observational unit. One-minute personal PM_2.5_ measurements were aggregated for each trip and matched with trip duration, distance, participant, season, and concurrent background concentration. Minute-level observations were not treated as independent statistical units. Results were summarized for the complete dataset and stratified descriptively by participant and season. Because most exposure and dose distributions were right-skewed, medians and interquartile ranges (IQRs) were used as the primary descriptive statistics, supplemented by arithmetic means and standard deviations where appropriate. No population-level inferential comparisons were performed because the dataset comprised repeated trips made by only two participants. Only trips with valid personal PM_2.5_, duration, distance, and corresponding background data were included. Missing measurements were not imputed. Data preparation and trajectory processing were performed in Python (version 3), including the GeoPy library. Dose, relative-risk, and Monte Carlo calculations were conducted in Microsoft Excel MS Office 365, 2019 (Microsoft Corporation, Redmond, WA, USA), with 10,000 realizations generated per trip for the variable-ventilation scenarios, while R programming language was used for data visualization. The calculation workbook documents the input audit and central formulas included in Supplementary File. Table S1 supplies the trip inputs, executable Python script (File S1) and uncertainty summaries for all three ventilation models, together with the humidity-stratified results (Table A1). The original trajectory-processing procedure is described separately in Section 2.2.

### 2.8. Joint Uncertainty and Deterministic Sensitivity Analyses

We propagated epidemiological parameter uncertainty on the log(*RR*) scale. For physical activity, the normal distribution had mean ln(0.87) and standard error [ln(0.91) − ln(0.83)]/(2 × 1.96). For PM_2.5_, the corresponding values were ln(1.095) and [ln(1.127) − ln(1.064)]/(2 × 1.96) per 10 µg/m^3^. These approximations use the reported confidence limits [50,51].

In each of 10,000 iterations, one physical-activity coefficient and one PM_2.5_ coefficient were drawn and shared across all 162 trips. Coefficients were independent of each other and of ventilation draws. We used NumPy Generator PCG64 with seed 4,539,044, drawing the coefficient arrays first, and then the B1 and B2 ventilation arrays in trip order. Model A held ventilation fixed while propagating both coefficients; B1 and B2 additionally varied ventilation independently for each trip. *RR_PA_* was obtained by exponentiating the sampled activity coefficient multiplied by the transformed activity dose. *RR_PM_* used the sampled pollution coefficient and the simulated dose-equivalent increment, and *RR_NET_* was their trip-specific product.

For every trip, we calculated the median, 2.5th and 97.5th percentiles of each simulated risk component and *NetEffect* and the proportion of realizations with *RR_NET_* < 1. In each iteration we also calculated the median across the 162 trips and then summarized the distribution of 10,000 dataset medians. We did not pool all trip-by-iteration realizations. The 2.5th–97.5th percentiles are termed a 95% uncertainty interval (UI), not a confidence interval for a population or a group median.

Without a device-specific calibration or measurement-error distribution, sensor error was not assigned a probability distribution. Instead, personal PM_2.5_ concentrations were multiplied by 0.90, 1.00 or 1.10, with stationary reference concentrations unchanged. The full calculation was repeated using central Model A and the probabilistic models. Identical coefficient and ventilation draws were reused across sensor factors to permit paired comparisons. The ±10% range is illustrative and must not be interpreted as a device-specific calibration interval, confidence interval or bound on actual measurement error.

For Model A, the concentration multiplier that gives Δ*C_eq_* = 0 was calculated as the counterfactual dose divided by the baseline cycling dose. A trip was classified as crossing zero within ±10% if this multiplier lay between 0.90 and 1.10. At central coefficients, the break-even dose-equivalent increment was ln(1/*RR_PA_*) divided by [ln(1.095)/10]. The corresponding cycling dose was obtained by adding the counterfactual dose to that increment multiplied by (7 × 11.92)/5; dividing by trip duration and cycling ventilation gave the personal PM_2.5_ break-even concentration. Substitution into the original equations verified *RR_NE_*_T_ = 1. Only personal concentration was varied, so these are trip-specific modelled thresholds under fixed assumptions.

To examine the available RH information, we used descriptive strata <60%, 60 to <80% and ≥80% based on trip-average RH recorded at the mobile laboratory. These exploratory cut-offs are not device-validation thresholds. We reported counts, personal concentration, Δ*C_eq_* and *RR_NET_* for the 150 complete records, with missing-RH trips separately. No calibration equation or inferential test was derived from this spatially separated comparison.

## 3. Results

### 3.1. Real-World PM_2.5_ Exposure During Urban Cycling

**Figure 2:**
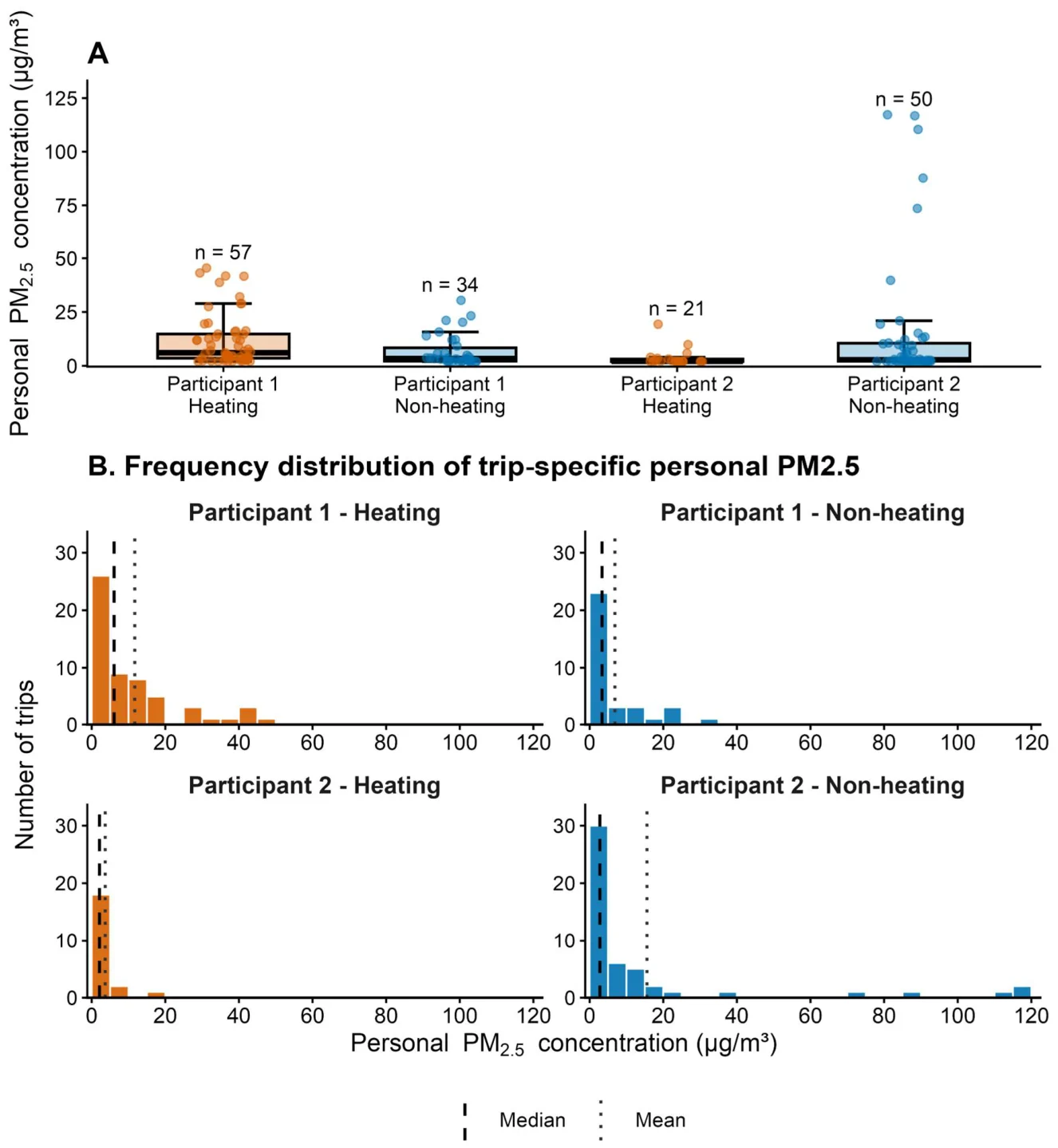
Distribution of trip-specific mean personal PM_2.5_ concentrations measured during cycling, stratified by participant and season. (**A**) Boxplots with individual trip-level observations; boxes represent the interquartile range and horizontal lines indicate medians. (**B**) Frequency distributions of trip-specific mean personal PM_2.5_ concentrations; dashed vertical lines indicate medians and dotted vertical lines indicate arithmetic means.

A total of 162 real-world cycling trips were included in the analysis, comprising 78 trips during the heating season and 84 during the non-heating season. Mean personal PM_2.5_ concentrations were 9.54 ± 11.13 µg/m^3^ during the heating season and 12.05 ± 23.99 µg/m^3^ during the non-heating season. Because the distributions were strongly right-skewed, the corresponding medians were considerably lower, reaching 4.13 µg/m^3^ (IQR: 2.28–12.44) and 3.06 µg/m^3^ (IQR: 2.11–10.30), respectively. This substantial difference between the mean and median reflects the occurrence of a limited number of relatively high-exposure cycling trips, particularly during the non-heating period. Figure 2 displays the observations without excluding high values. Table 1 presents participant-specific descriptive summaries. Participant 1 had a higher median personal concentration in the heating season (6.07 µg/m^3^) than in the non-heating season (3.36 µg/m^3^). For Participant 2, the corresponding medians were 2.12 and 2.78 µg/m^3^. The non-heating-season mean for Participant 2 was 15.58 ± 30.11 µg/m^3^, reflecting a small number of high-concentration trips. These comparisons describe the monitored records and do not estimate population-level participant, sex or seasonal effects.

The cycling records are a subset of the previously published multimodal dataset [30]. Figure 2 is a new plot of that subset, further separated by participant; it is not new exposure data. Differences among trips could reflect route and timing, intermittent local sources, meteorology or optical sensor response. The dataset does not separate these mechanisms.

The observed trip-specific patterns suggest that stationary background PM_2.5_ concentrations may not fully capture the short-term exposure encountered by cyclists along individual routes. However, the present two-participant dataset does not permit population-level inference regarding this relationship.

The ventilation assumptions substantially affected the magnitude of the estimated inhaled dose. Median Model B1 doses were lower than the corresponding fixed-ventilation Model A estimates in both seasons, whereas Model B2 produced intermediate estimates. These differences reflect the ventilation assumptions applied in the respective models rather than changes in the measured exposure. The comparison of Models A, B1, and B2 is therefore interpreted descriptively as a sensitivity analysis of the dependence of estimated inhaled dose on pulmonary ventilation assumptions.

### 3.2. From Exposure to Inhaled Dose

Inhaled PM_2.5_ dose was calculated for each of the 162 cycling trips using measured personal exposure, individual trip duration, and ventilation assumptions.

The inhaled-dose estimates varied with the assumed ventilation. Model A used fixed reference rates; B1 and B2 used independent trip-specific draws from the stipulated uniform ranges. Dose remains a modelled quantity in all scenarios.

Across all trips, the absolute inhaled-dose estimates varied considerably according to the ventilation scenario. In the heating season, the arithmetic mean dose was 23.13 ± 33.49 µg/trip for Model A, 8.74 ± 12.06 µg/trip for Model B1, and 14.37 ± 20.46 µg/trip for Model B2. Corresponding values during the non-heating season were 20.37 ± 38.91, 11.17 ± 24.60, and 14.48 ± 29.24 µg/trip, respectively. Because the dose distributions were strongly skewed, median values were lower as follows: 10.59, 3.88, and 6.47 µg/trip for Models A, B1, and B2 during the heating season and 6.11, 2.97, and 4.38 µg/trip, respectively, during the non-heating season.

### 3.3. Health Benefits of Cycling

The modelled relative risks and corresponding health benefits of the observed cycling duration and standardized scenarios are presented in Table 2. Assuming cycling on five working days per week, the median observed daily cycling duration was 33.0 min/day (IQR: 19.0–47.0 min/day), corresponding to a weekly physical-activity dose (*PA* Dose) of 18.70 MET-h/week (IQR: 10.76–26.63 MET-h/week). The central median *RR_PA_* was 0.836, equivalent to a modelled relative risk reduction of 16.4%. Table 2 reports the observed-duration scenario and standardized daily durations. Its endpoint bounds are transformations of the source epidemiological confidence limits, not confidence intervals estimated from the two commuters.

**Figure 3:**
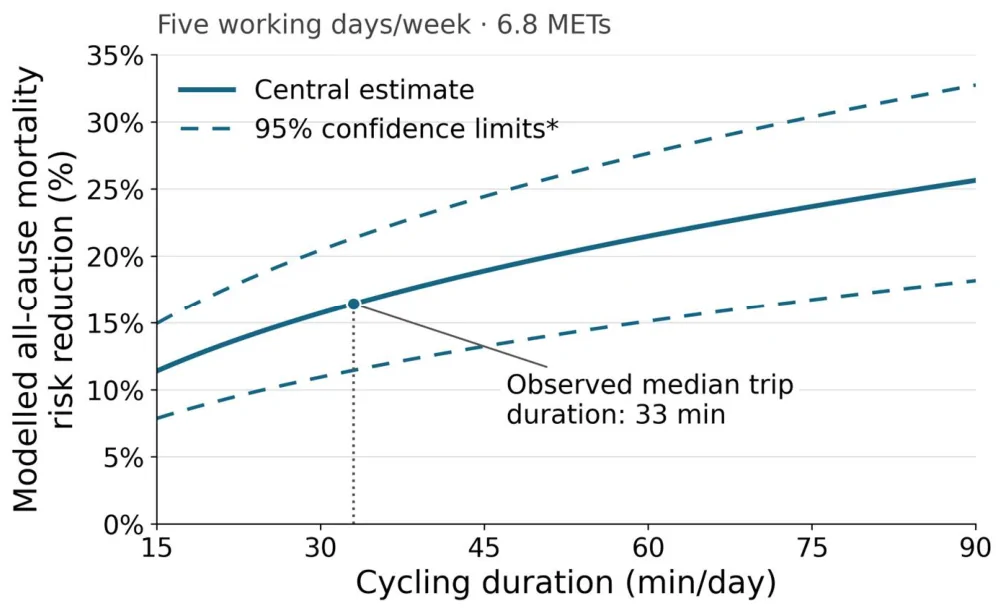
Modelled reduction in all-cause mortality risk associated with cycling for 15–90 min/day on five working days per week, * 95% limits reflect the physical-activity coefficient only.

For standardized daily durations of 15–90 min, central *RR_PA_* decreased from 0.886 to 0.743. Figure 3 shows the complete assumed activity curve and the observed median duration. The shaded limits vary the epidemiological coefficient while holding frequency, intensity and the power transformation fixed. The figure excludes the PM_2.5_ component and should not be interpreted as an observed response to cycling.

### 3.4. PM_2.5_-Related Health Risk

Trip-specific PM_2.5_-related risk estimates for the overall dataset and stratified by season and participant are summarized in Table 3. Across 162 cycling trips, the median additional equivalent PM_2.5_ exposure, Δ*C_eq_*, was 0.2 µg/m^3^ (IQR: −0.004–0.817 µg/m^3^). This corresponded to a median modelled *RR_PM_* of 1.00182, with propagated lower and upper limits of 1.00124 and 1.00239, respectively. The corresponding median PM_2.5_-related risk increase was 0.182% (propagated limits: 0.124–0.239%).

IQRs describe variation among the observed trip scenarios. Negative increments indicate a lower estimated cycling dose than the selected resting-reference dose; they do not imply a protective biological effect of PM_2.5_. The median *RR_PM_* was 1.00289 in the heating season and 1.00086 in the non-heating season. Participant-specific medians were 1.00321 and 1.00024 for Participants 1 and 2. There were 120 positive increments and 42 negative increments. The negative values arise from the counterfactual comparison and should not be interpreted as a benefit of PM_2.5_ exposure. Using the historical coefficient *RR* = 1.07 per 10 µg/m^3^ [4] gave a median *RR_PM_* of 1.00135.

### 3.5. Central Net Relative Risks

The combined benefit–risk model yielded *RR_NET_* < 1 for all 162 trip-based scenarios under the central modelling assumptions (Table 4). Across all trips, the median physical-activity-related relative risk, *RR_PA_*, was 0.836, whereas the median PM_2.5_-related relative risk, *RR_PM_*, was 1.00182. The trip-specific combination of these effects resulted in a median *RR_NET_* of 0.840 (IQR: 0.815–0.879), corresponding to a modelled median net reduction in relative all-cause mortality risk of 16.0% under the specific habitual behaviour.

All results are descriptive model outputs for the recorded trip patterns. Median *RR_NET_* values were 0.831 and 0.855 for the heating and non-heating seasons, and 0.824 and 0.861 for Participants 1 and 2. These patterns reflect the joint effects of duration, concentration, reference exposure and ventilation assumptions. They do not estimate seasonal or participant effects in a wider population. Replacing the primary PM_2.5_. coefficient with *RR* = 1.07 per 10 µg/m^3^ gave a median *RR_NET_* of approximately 0.8393. This coefficient-only sensitivity produced little change in the central median, but does not resolve other uncertainties in exposure or model structure.

### 3.6. Joint Uncertainty Sensor Sensitivity and Break-Even

At the baseline sensor factor, joint coefficient uncertainty with fixed Model A ventilation gave a median dataset *RR_NET_* of 0.8395 (95% UI 0.7918–0.8920). For B1 and B2, the corresponding summaries were 0.8348 (0.7876–0.8862) and 0.8363 (0.7888–0.8883). In every model and sensor scenario, the dataset median was below 1 in all 10,000 iterations.

**Figure 4:**
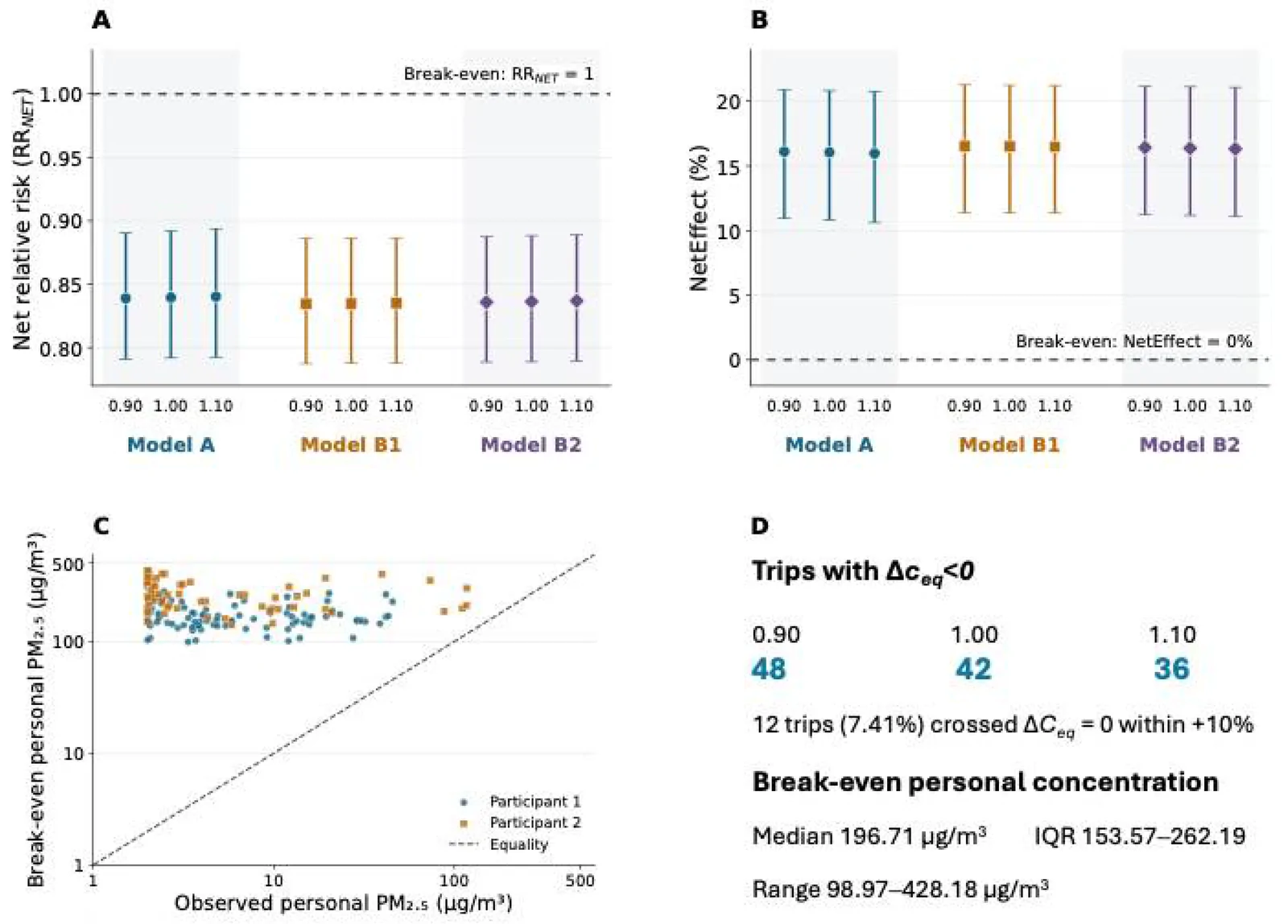
Break-even concentrations: (**A**) net relative risk; (**B**) NetEffect; (**C**) observed vs. break-even concentrations; (**D**) sensitivity and break-even summary.

In deterministic Model A, the −10%, baseline and +10% sensor scenarios gave median *RR_NET_* values of 0.83975, 0.84000 and 0.84125. Negative Δ*C_eq_* counts were 48, 42 and 36. Twelve trips (7.41%) crossed Δ*C_eq_* = 0 somewhere within the ±10% range, while no central *RR_NET_* estimate reached 1. Figure 4 summarizes these illustrative scenarios.

The median Model A personal PM_2.5_ break-even concentration was 196.71 µg/m^3^ (IQR 153.57–262.19; range 98.97–428.18). The median required concentration multiplier was 43.39 (IQR 16.92–102.86), with a minimum of 1.79. No break-even multiplier fell within 0.90–1.10. Figure 4 compares the observed concentrations with these modelled values. They should not be interpreted as safe exposure limits or thresholds applicable to other populations.

## 4. Discussion

### 4.1. Balance Between the Physical-Activity Benefit and PM_2.5_-Related Risk

The principal finding of this study is that, under the real-world cycling conditions observed in Gliwice, the modelled health benefit associated with physical activity substantially exceeded the estimated mortality risk attributable to additional PM_2.5_ exposure.

The central model produced a favourable PM_2.5_ physical-activity balance for the 162 recorded trip patterns when each was extrapolated to habitual cycling five days per week. The component medians were *RR_PA_* = 0.836 and *RR_PM_* = 1.00182, and the median of their trip-specific products was *RR_NET_* = 0.840. These numbers arise from the specified exposure and activity functions. They are not evidence of measured mortality reduction in the two participants.

Joint propagation of coefficient uncertainty with fixed ventilation gave a dataset-median *RR_NET_* of 0.8395 (95% UI 0.7918–0.8920). The corresponding B1 and B2 summaries were 0.8348 (0.7876–0.8862) and 0.8363 (0.7888–0.8883). The median remained below 1 in every simulated iteration. This conditional finding does not mean that the outcome is certain: individual simulated values can exceed 1, and uncertainty from model form, sensor calibration and transferability is not fully represented.

These findings are consistent with previous health-impact assessments showing that the benefits of cycling generally outweigh air-pollution-related risks [1,2,3,4,5,6,7]. De Hartog et al. [1] and Rojas-Rueda et al. [2] demonstrated that the expected gains from increased physical activity were substantially greater than the adverse effects associated with air pollution exposure. Tainio et al. [4,41] similarly concluded that the benefits of active travel would be negated only under unusually high PM_2.5_ concentrations or very prolonged exposure. For 30 min of cycling per day, their estimated tipping and break-even points occurred at approximately 95 and 160 µg/m^3^, respectively. These values should not be transferred directly to Gliwice because the present study employed measured personal exposure, a dose-equivalent concentration and an updated mortality concentration–response function. Nevertheless, they provide useful context indicating that the PM_2.5_ levels required to completely offset the benefits of cycling are substantially higher than those typically encountered during the cycling trips included in the present study.

### 4.2. Importance of Real-World Personal Exposure Assessment

An important contribution of the present study is the use of personal PM_2.5_ measurements collected during routine cycling trips rather than an assumed relationship between urban-background concentration and cyclist exposure. Tainio et al. [4,41] multiplied background PM_2.5_ concentrations by a factor of 2.0 to approximate exposure during active travel. Although appropriate in the absence of personal measurements, such an approach cannot account for route-specific and short-term exposure variability. In the present analysis, this conversion factor was unnecessary because PM_2.5_ concentrations were measured directly within the cyclist’s breathing environment.

The observed personal exposure did not reproduce a uniform seasonal pattern. The direction of the seasonal difference varied between the two participants, despite the generally higher background PM_2.5_ concentrations recorded at stationary monitoring sites during the heating season. This result confirms that a stationary urban-background concentration should not automatically be treated as a direct surrogate for exposure experienced by cyclists. Personal exposure depends on route selection, proximity to traffic, intersections, street geometry, local sources and the temporal coincidence between a cyclist’s journey and short-lived pollution events [22,23,24,25,26,27,28,29,30,31]. Recent mobile-monitoring and low-cost-sensor studies have similarly demonstrated considerable spatial and temporal variation in exposure along urban cycling routes [28,29,31,52,53].

The distinction between personal concentration and inhaled dose is also essential. A cyclist may encounter a relatively low PM_2.5_ concentration while still inhaling a relevant particle mass because physical activity increases pulmonary ventilation. Conversely, a higher concentration encountered during a short trip may produce a smaller inhaled dose than a lower concentration experienced for a longer period. Studies comparing transport modes and cycling conditions have repeatedly shown that pollutant uptake depends jointly on concentration, exposure duration and respiratory ventilation [15,16,17,18,19,32,33,49,54]. The present results support this dose-based interpretation: seasonal and participant-specific differences in PM_2.5_-related risk reflected the combined effects of personal concentration, trip duration, assumed ventilation and the corresponding non-cycling background dose rather than concentration alone.

The sign of a small dose-equivalent increment is particularly sensitive to measurement and counterfactual assumptions. At baseline, 42 increments were negative. Within the illustrative ±10% personal-concentration range, 12 trips changed sign. In B1, the dataset median *RR_PM_* was slightly below 1 because lower ventilation often produced a cycling dose below the selected resting-reference dose. This is a comparison between exposure scenarios, not a biologically protective effect of particles.

### 4.3. Seasonal and Participant-Specific Patterns

The PM_2.5_-related risk contribution was greater during the heating season than during the non-heating season. Nevertheless, the median net benefit was also greater during the heating season: 16.9% compared with 14.5%. This apparently counterintuitive result can be explained by the longer median cycling duration during the heating season, which increased the weekly physical-activity dose. Although longer cycling also increased inhaled PM_2.5_ dose, the additional physical-activity benefit was considerably larger than the corresponding increase in PM_2.5_-related risk.

This finding illustrates why decisions concerning active travel should not be based on pollutant concentration alone. Discouraging cycling whenever pollution is elevated may reduce immediate exposure but may simultaneously remove a substantially larger long-term physical-activity benefit. Giallouros et al. [5] demonstrated that systematically restricting cycling and walking on high-pollution days could produce adverse long-term health consequences through reduced physical activity. This does not mean that exposure reduction is unnecessary. Rather, it supports interventions that preserve physical activity while reducing exposure, including separation of cycling infrastructure from heavily trafficked roads, selection of lower-exposure routes and avoidance of pollution hotspots [22,23,24,25,26,27,28,29,34].

Participant 1 also showed a greater PM_2.5_-related risk than Participant 2 but simultaneously obtained a greater net health benefit. The difference was associated primarily with a longer median cycling duration and, therefore, a higher physical-activity dose. Differences in the assumed ventilation rates additionally affected the calculated inhaled doses. Because participant identity and sex were completely confounded and only one male and one female cyclist were included, these results cannot be interpreted as evidence of population-level sex differences. They describe two individual commuting patterns under the ventilation assumptions used in the models.

### 4.4. Comparison with Epidemiological Evidence

The present results are broadly consistent with epidemiological evidence suggesting that physical activity remains beneficial across a wide range of air-pollution conditions. Prospective studies conducted in China have indicated that high long-term PM_2.5_ exposure may attenuate some cardiovascular benefits of active commuting [37,38]. In contrast, evidence from the UK suggests that active commuting can remain protective at the lower pollution levels typically encountered in European settings [39]. Systematic and mapping reviews similarly conclude that air pollution may reduce the magnitude of some physical-activity benefits but does not support a universal pollution threshold above which physical activity becomes harmful [40,41].

Recent analyses of physical activity and mortality also indicate that habitual exercise is generally associated with lower mortality risk in polluted environments, although the degree of protection may diminish as long-term PM_2.5_ exposure increases [42,43,44,46,47]. The Gliwice results fit within this broader body of evidence: PM_2.5_ introduced a measurable adverse contribution, but this contribution was insufficient to negate the much larger benefit attributed to habitual cycling.

The present assessment concerns modelled long-term all-cause mortality effects and should not be interpreted as an estimate of acute responses to an individual cycling trip. Short-term respiratory or cardiovascular responses, symptoms in susceptible individuals and effects of brief high-exposure episodes were not evaluated. Accordingly, the favourable long-term balance does not preclude recommending exposure-reduction measures, particularly for people with asthma, cardiovascular disease or other conditions that may increase sensitivity to air pollution.

Acute symptoms, susceptible groups and short pollution peaks fall outside the model. A favourable long-term scenario cannot be interpreted as advice that cycling is safe for every person during every pollution episode.

### 4.5. Strengths and Limitations

The main strength of the study is the integration of one-minute personal PM_2.5_ measurements, GPS-based cycling trajectories, and observed trip durations with modelled cycling-specific ventilation, inhaled dose, and quantitative physical-activity benefit–risk modelling. Unlike previous assessments based primarily on ambient concentrations and assumed cyclist-to-background ratios, the present approach retained real-world trip-to-trip variability. The use of the same endpoint—all-cause mortality—for both the physical-activity and PM_2.5_ components also enabled the two effects to be combined within a consistent relative-risk framework. The sensitivity analyses showed that ventilation assumptions affected the magnitude of the estimated inhaled dose, whereas the use of an alternative PM_2.5_ concentration–response coefficient did not change the overall net-health interpretation.

Several limitations must nevertheless be considered. First, the dataset comprised repeated trips made by only two participants. The results characterize the observed commuting patterns but cannot be generalized to the wider population of cyclists in Gliwice or used to infer sex-related differences. Personal PM_2.5_ concentrations were measured using portable optical low-cost sensors. Published Flow 2 performance assessments were conducted under conditions that differed from the present mobile measurements and did not provide device-specific bias, RMSE or MAE values for the units used here. No quantitative RH correction was applied, although optical particle measurements may be affected by relative humidity and aerosol properties. This limitation is particularly relevant because the median Δ*C_eq_* was only 0.200 µg/m^3^ and 42 trips produced negative values. Sensor error could therefore affect the sign and magnitude of Δ*C_eq_* for trips with values close to zero. Sensor-related error was examined using illustrative deterministic ±10% personal-concentration scenarios rather than a device-specific probability distribution. The joint uncertainty intervals incorporate uncertainty in both epidemiological coefficients and, for Models B1 and B2, the stipulated ventilation distributions.

Pulmonary ventilation was not measured directly during each trip. The fixed-ventilation model and Monte Carlo scenarios were based on values reported in previous studies and may not fully represent actual ventilation associated with changes in speed, effort, elevation, weather or individual fitness.

Using trip-average concentration and ventilation also omits their temporal covariance. The resulting dose can be underestimated when higher concentrations coincide with higher ventilation or overestimated when the reverse occurs. Without synchronized concentration and ventilation measurements, the magnitude and direction of this effect cannot be quantified for these trips.

The health assessment assumed that the duration of each observed trip represented habitual cycling repeated on five working days per week. Changes in commuting frequency, seasonal cycling behaviour or total baseline physical activity were not available. The physical-activity model therefore estimates the long-term benefit of a hypothetical habitual pattern corresponding to each observed trip rather than the acute benefit of that journey. The same physical-activity dose–response coefficient was applied to both participants without adjustment for age, fitness, health status or activity undertaken outside commuting.

The conversion of additional inhaled dose into a long-term dose-equivalent concentration assumes proportionality between inhaled PM_2.5_ mass and the mortality risk reported in studies of ambient long-term exposure. It does not account for differences in particle composition or toxicity. The joint Monte Carlo uncertainty intervals describe propagated parameter uncertainty conditional on the recorded trip patterns and specified model assumptions; they are not population-level confidence intervals and do not quantify structural model uncertainty. Finally, the assessment considered PM_2.5_ and physical activity only. Other traffic-related pollutants, ultrafine particles, black carbon, noise and road-traffic injuries were outside the scope of the analysis. The results should therefore be interpreted as a targeted PM_2.5_–physical-activity comparison rather than a complete health-impact assessment of cycling.

### 4.6. Public-Health Interpretation

Under the real-world conditions represented by the monitored cycling trips in Gliwice, the estimated PM_2.5_-related mortality risk was substantially smaller than the physical-activity benefit. The results are therefore consistent with the broader evidence supporting the promotion of habitual cycling as a public-health and sustainable-transport measure. At the same time, the evidence of variable personal exposure indicates that policies should aim to make cycling cleaner rather than discourage it. Priority measures include reducing traffic emissions, expanding cycling infrastructure separated from major roads, identifying low-exposure routes and improving the availability of real-time information on local air quality.

Future studies should recruit more diverse participants and routes, collect collocated reference measurements and assess ventilation or exertion alongside baseline activity. Evaluating lower-exposure routes and infrastructure would require actual comparisons of those alternatives. The potential advantage is maintaining activity while reducing inhaled pollution; the present study cannot quantify implementation trade-offs, accident risk or benefits for susceptible groups.

## 5. Conclusions

This secondary analysis of 162 trips by two commuters evaluated a hypothetical habitual-cycling pattern against resting at a concurrent stationary reference concentration. Under central assumptions, the median *RR_NET_* was 0.840. The result is a modelled PM_2.5_–physical-activity balance, not an observed or population-level mortality effect.

Joint coefficient uncertainty and the alternative ventilation scenarios retained dataset-median *RR_NET_* values below 1 in the simulated distributions. Illustrative ±10% personal-concentration changes altered the sign of Δ*C_eq_* for 12 trips without producing *RR_NET_* ≥ 1 under central coefficients. The median trip-specific break-even concentration was 196.7 µg/m^3^, conditional on fixed activity, ventilation and counterfactual assumptions.

The contribution is a reproducible framework showing which conclusions persist within specified scenarios and which quantities, particularly small dose increments, are sensitive to assumptions. Uncalibrated optical measurements and the two-person sample prevent wider quantitative interpretation.

Larger studies with sensor validation, measured exertion and explicit alternatives are required before translating these numerical estimates into population or transport-policy conclusions.

## Figures and Tables

**Table 1:** Descriptive characteristics of the monitored cycling trips by participant and season.

Participant	Season	*n* Trips	PM_2.5_ Median (IQR) µg/m^3^	PM_2.5_ Mean ± SD	Trip Duration Median (IQR), min
Participant 1	Heating	57	6.07 (3.55–14.78)	11.70 ± 12.12	44.00 (32.00–50.98)
Participant 1	Non-heating	34	3.36 (2.16–8.36)	6.87 ± 7.38	33.52 (22.37–54.52)
Participant 2	Heating	21	2.12 (2.00–3.14)	3.67 ± 4.04	21.02 (14.00–32.02)
Participant 2	Non-heating	50	2.78 (2.11–10.50)	15.58 ± 30.11	24.01 (15.99–40.01)

**Table 2:** Modelled relative risk of all-cause mortality and the corresponding health benefits associated with cycling on five working days per week.

Cycling Scenario	Cycling Duration (min/Day)	*PA* Dose (MET-h/Week)	*RR_PA_* (Parameter Bounds)	Risk Reduction (Parameter Bounds), %
Observed trips—median (IQR)	33.0 (19.0–47.0)	18.70 (10.76–26.63)	0.836 (0.786–0.886)	16.4 (11.4–21.4)
15 min/day	15	8.50	0.886 (0.850–0.921)	11.4 (7.9–15.0)
30 min/day	30	17.00	0.843 (0.795–0.891)	15.7 (10.9–20.5)
45 min/day	45	25.50	0.811 (0.755–0.868)	18.9 (13.2–24.5)
60 min/day	60	34.00	0.785 (0.723–0.849)	21.5 (15.1–27.7)
90 min/day	90	51.00	0.743 (0.673–0.818)	25.7 (18.2–32.7)

**Table 3:** Modelled PM_2.5_-related relative risks under the primary fixed-ventilation model.

Group	Trips, *n*	Δ*C_eq_*, Median (IQR), µg/m^3^	*RR_PM_*, Median * (Propagated Limits)	Risk Increase, %	*RR_Tainio_*, Median	Negative Δ*C_eq_*, *n* (%)
All trips	162	0.200 (−0.004–0.817)	1.00182 (1.00124–1.00239)	0.182 (0.124–0.239)	1.00135	42 (25.9)
Heating	78	0.318 (0.049–0.852)	1.00289 (1.00197–1.00380)	0.289 (0.197–0.380)	1.00215	11 (14.1)
Non-heating	84	0.094 (−0.034–0.811)	1.00086 (1.00059–1.00113)	0.086 (0.059–0.113)	1.00064	31 (36.9)
Participant 1	91	0.353 (0.104–0.990)	1.00321 (1.00219–1.00423)	0.321 (0.219–0.423)	1.00239	14 (15.4)
Participant 2	71	0.027 (−0.034–0.330)	1.00024 (1.00017–1.00032)	0.024 (0.017–0.032)	1.00018	28 (39.4)

* *RR_PM_* estimates were calculated using trip-specific inhaled doses obtained from Model A.

**Table 4:** Net relative risk of all-cause mortality and net health benefit associated with cycling.

Group	*RR_NET_*, Median (IQR)	Net Health Benefit, Median (Uncertainty Limits), %	Robust Net Benefit *, *n*/*N* (%)
All trips	0.840 (0.815–0.879)	16.0 (10.5–21.1)	160/162 (98.8%)
Heating season	0.831 (0.810–0.866)	16.9 (11.2–22.5)	78/78 (100%)
Non-heating season	0.855 [0.823–0.884]	14.5 (9.9–19.4)	82/84 (97.6%)
Participant 1	0.824 [0.804–0.854]	17.6 (11.7–23.4)	91/91 (100%)
Participant 2	0.861 [0.833–0.891]	13.9 (9.6–18.4)	69/71 (97.2%)

* IQR describes the distribution of central *RR_NET_* estimates across trips. Uncertainty limits were propagated from the 95% confidence limits of the physical-activity and PM_2.5_ concentration–response functions and should not be interpreted as confidence intervals for the group median. A net benefit under combined epidemiological coefficient bounds was defined as an upper uncertainty limit of *RR_NET_* < 1. Participant-specific results are descriptive and should not be interpreted as general sex-related effects.

## Data Availability

The original exposure dataset was described by Mainka et al. [30]. The data supporting the findings of this study are openly available in the RepOD research data repository at https://doi.org/10.18150/VDIMJT.

## Supplementary Materials

The following supporting information can be downloaded at: https://www.mdpi.com/article/10.3390/toxics14090836/s1, File S1: Reproducibility code for the joint uncertainty and sensitivity analyses used in the manuscript on cycling-related personal PM_2.5_ exposure; Table S1: Cycling PM_2.5_ benefit–risk modelling: input data, model parameters, joint uncertainty propagation, sensor sensitivity and break-even analyses.

## Appendix A

RH was recorded at the stationary mobile laboratory and is not a sensor-inlet measurement. The exploratory strata below do not identify or correct instrument bias. All statistics use central Model A. Twelve missing-RH records remain in the principal dataset. Heating-season trips are numbered 29, 25, 21 and 3 in the four rows, respectively; Participant 1 contributed 41, 21, 19 and 10. This imbalance illustrates confounding in a simple RH comparison. Missing-RH Trip_ID values were 14, 15, 16, 68–74, 92 and 132. The metadata source was the processed bicycle sheet, column BP, linked by the audited trip mapping.

**Table A1:** Descriptive humidity strata.

RH Stratum	*n*	PM_2.5_ Median (IQR) µg/m^3^	Δ*C_eq_* Median (IQR) µg/m^3^	Median *RR_NET_*	Negative Δ*C_eq_ n*
<60%	62	3.66 (2.30–11.64)	0.229 (0.031–0.783)	0.8321	12
60 to <80%	46	3.20 (2.08–11.38)	0.073 (−0.031–0.675)	0.8504	14
≥80%	42	4.14 (2.11–10.02)	0.089 (−0.032–0.601)	0.8478	16
Missing RH	12	5.47 (3.76–20.99)	0.779 (0.488–2.960)	0.8518	0
